# A Case of Loss of Consciousness Due to Thrombosis of the Inferior Vena Cava and Portal Vein

**DOI:** 10.7759/cureus.41306

**Published:** 2023-07-03

**Authors:** Shintaro Takeuchi, Tatsuya Hayakawa

**Affiliations:** 1 Emergency Department, Seirei Mikatahara General Hospital, Hamamatsu, JPN; 2 Emergency Medicine Department, Seirei Mikatahara General Hospital, Hamamatsu, JPN

**Keywords:** intestinal necrosis, deep vein thrombosis, superior mesenteric vein thrombosis, portal vein thrombosis, inferior vena cava thrombosis

## Abstract

We present a case where intraperitoneal venous thrombosis was difficult to treat. It is difficult to suspect intraperitoneal venous thrombosis in patients who have visited the hospital due to loss of consciousness, and it is necessary to administer anticoagulants early for treatment and to determine the appropriate timing for surgical intervention.

The patient was a 78-year-old male who independently performed his daily activities. On the day of admission, he lost consciousness and was brought to our hospital. Computed tomography (CT) angiography revealed thrombi from the inferior vena cava and portal vein to the superior mesenteric vein, and the patient was started on anticoagulant therapy. The CT angiography images on day 7 of the illness revealed that the thrombus in the superior mesenteric vein expanded to the caudal side. Intestinal necrosis occurred on day 22 of the illness, and emergency laparotomy was performed. The chosen course of treatment was successful, and the patient was discharged on the 48th day.

## Introduction

The simultaneous occurrence of extensive thrombosis from the inferior vena cava and portal vein to the superior mesenteric vein is uncommon. We encountered a rare case in which the patient was brought to emergency due to loss of consciousness, with difficulties faced in the treatment, but could be discharged without any major sequelae on day 48 of admission, and herein report the case.

## Case presentation

The patient is a 78-year-old male with a past medical history of hypertension and diabetes. His main complaint was loss of consciousness. His social history was significant for no smoking, occasional drinking, and working as a bus driver. Three days before admission in September 2020, he experienced stomach bloating. When he visited a nearby clinic two days before hospitalization, he was asked about his intestinal gas retention. He was living at home until the day of admission. On the morning of the day of admission, his family members found him unconscious on the bed and call for an ambulance, and he was brought to our hospital.

On presentation, the patient had a Glasgow coma scale score of 3, respiratory rate of 12 breaths/min, SpO_2_ level of 99% (oxygen mask 5L), heart rate of 103 beats/min, blood pressure of 120/74 mmHg, bilateral pupil diameters of 3 mm, and a body temperature of 36.4℃. There was a dull light reflex in both eyes, the abdomen was soft and non-tender, and no bowel peristalsis hyperattenuation was observed.

Test findings on presentation (Table 1) were as follows: respiratory alkalosis, hypoalbuminemia, hyperammonemia, hyponatremia, hyperkalemia, and increased C-reactive protein levels, as well as elevated white blood cell count, fibrinogen degradation products (FDP) level, and D-dimer level. Blood culture results were negative (clarified after five days), hepatitis virus markers were negative, no abnormality was noted in other cerebrospinal fluid tests, and results were negative for various tumor markers.

**Table 1:** Laboratory data on admission

Laboratory parameters	Results	Reference range
pH	7.577	7.35-7.45
PaCO_2_	24.9 mmHg	35-45
PaO_2_	101.5 mmHg	80-100
HCO_3-_	22.7 mEq/L	22-26
Lactate	30.7 mg/dl	4.5-13.5
White blood cell count	13,140/μL	4.0-10.0
Hemoglobin	15.7 g/dL	12.0-15.0
Platelets	157,000/μL	150-400
Albumin	2.2 g/dL	3.8-4.9
Ammonia	343 μg/dL	30-80
Sodium	132 ｍEq/L	133-146
Potassium	5.6 mEq/L	3.5-5.3
C-reactive protein	28.8 mg/dL	0-0.3
Fibrinogen degradation products	75.5 μg/dL	0-0.5
D-dimer	51.7 μg/mL	0-1.0

**Figure 1:**
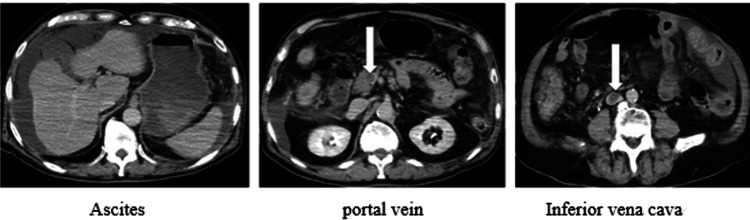
The abdominal CT images on day 1 of admission. Thrombi are observed at the tips of the arrows. CT, computed tomography

Abdominal contrast-enhanced computed tomography (CT) (Figure 1) revealed thrombi extending from the inferior vena cava and portal vein to the superior mesenteric vein, and ascites mainly around the liver.

On admission, sepsis due to intraperitoneal organ infection at an unknown site was suspected. The patient presented with loss of consciousness; therefore, tracheal intubation and ventilator management were started, and meropenem 1 g was administered. Because systolic blood pressure dropped to 70 mmHg immediately after admission to the intensive care unit, and hydrocortisone 200 mg IV and vasopressin were administered. On day 2 of admission, abdominal contrast-enhanced CT interpretation was performed by the radiology department, which revealed thrombi from the inferior vena cava and portal vein to the superior mesenteric vein, and the patient was started on heparin at 10,000 units/day. However, blood test results showed no abnormality in the coagulation-fibrinolytic system.

**Figure 2:**
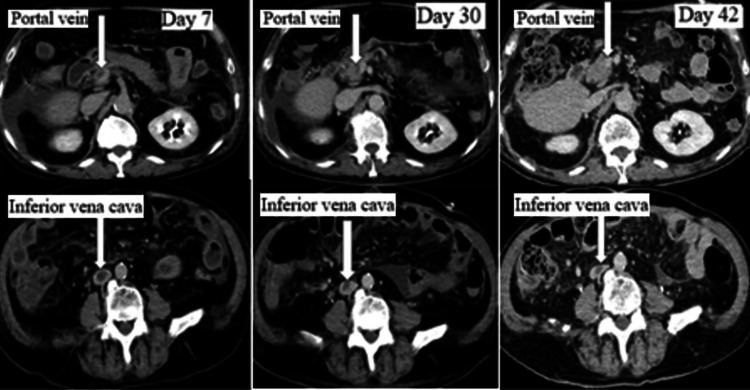
The contrast-enhanced CT images on day 7, day 30, and day 42. Thrombi are observed at the tips of the arrows. CT, computed tomography

On day 3 of the illness, as the serum ammonia level decreased to 86 μg/dL, the level of consciousness improved, and respiratory and circulatory conditions were stabilized. However, on day 7 of the illness, a consciousness disorder was observed and hypoxemia was confirmed; therefore, thoracoabdominal contrast-enhanced CT scanning was performed. As a result, pulmonary embolism could not be excluded. The thrombus in the superior mesenteric vein was expanding to the caudal side (Figure 2).

**Figure 3:**
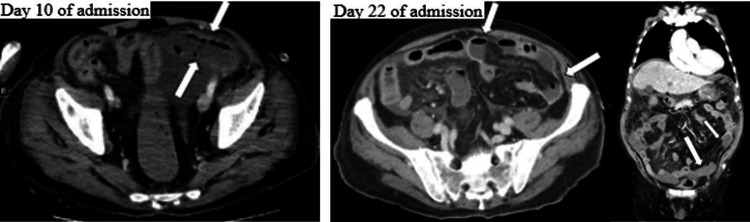
The contrast-enhanced CT images on day 10 and day 22. The tips of the arrows show low-contrast regions. CT, computed tomography

From days 9 to 11 of illness, because of concerns regarding decreased heparin activity due to decreased antithrombin (AT) levels, the AT Ⅲ formulation (Neuart; 1,500 units/day) was administered. On day 14 of the illness, the patient’s respiratory and circulatory conditions were stable, and he was conscious. On day 16 of the illness, abdominal contrast-enhanced CT scanning showed a shrinking tendency in all thrombi, but black stool appeared and blood test results revealed anemia, and, therefore, the patient was put on fasting and started on proton pump inhibitor administration and blood transfusion. On day 19 of the illness, upper gastrointestinal endoscopy was performed, but no upper gastrointestinal bleeding was confirmed. On day 22 of the illness, when abdominal contrast-enhanced CT scanning (Figure 3) was performed, intraperitoneal free gas images were obtained and small bowel perforation was suspected; therefore, emergency laparotomy was performed.

**Figure 4:**
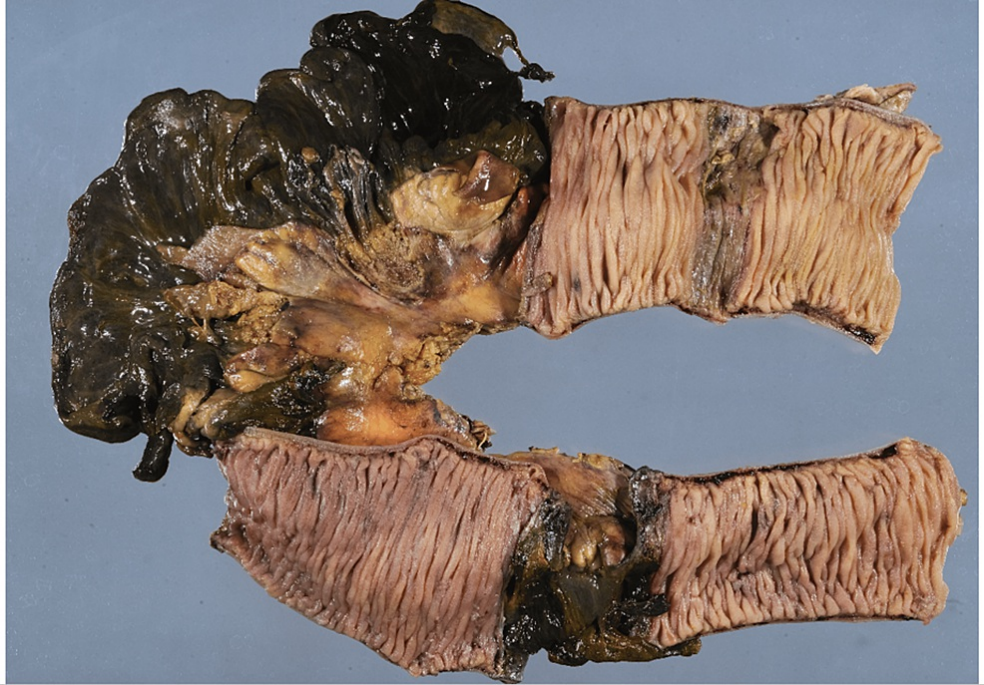
Part of the pathological tissues of the small bowel resection specimen. Macroscopically, the intestinal wall was necrotic and thinning transmurally, exhibiting a dark green tone. Histologically, the intestinal wall structure was lost at the necrotic site, and transmural purulent inflammation and granulation were observed. Thrombi were conspicuous in the intestinal membrane of the necrotic site, there were organized thrombi in places, and fat necrosis, perivascular/fatty lobular septal and intralobular/serosal surface fibrosis, and granulation are observed. Congestion, edema, hemosiderin deposition, and neutrophil infiltration at the non-necrotic site, and mainly purulent inflammation and granulation were observed at the boundary between the necrotic site and the non-necrotic site. No neoplastic lesion was observed.

Surgical findings showed that approximately 20 cm of the small bowel was necrotic on the anal side at around 50 cm from the ligament of Treitz. Then, after approximately 15 cm of the small bowel with a normal serosal surface, around 5 cm of small bowel necrosis was again observed. Cloudy ascites were observed in the surrounding area, and an abscess was observed in some areas. Two necrotic lesions were resected, totaling around 52 cm of the small bowel. Pathological findings revealed intravenous thrombi at the venule level in the mesentery of the necrotic site. In addition, fat necrosis, perivascular/fatty lobular septal and intralobular/serosal surface fibrosis, and granulation were observed, which was consistent with infarct images associated with the thrombi (Figure 4).

On day 25 of the illness, tube feeding and heparin administration were restarted, and the heparin administration duration was 23 days. Within this period, AT Ⅲ formulation was administered for 7 days (day 9 of illness to day 11 of illness, day 13 of illness, day 15 of illness, day 17 of illness, and day 29 of illness). Contrast-enhanced CT on day 30 of illness (Figure 2) revealed a shrinking tendency in the thrombi, and the condition improved until food consumption became possible. From day 32 of the illness, heparin was replaced with edoxaban at 60 mg/day. Thoracoabdominal contrast-enhanced CT on day 42 of the illness (Figure 2) revealed that the thrombi had almost disappeared. On day 44 of admission, the patient was discharged on an independent gait. On day 163 of admission, abdominal contrast-enhanced CT performed at the cardiology outpatient clinic confirmed the disappearance of thrombi; therefore, oral edoxaban administration was discontinued. As a result, no further examinations were required.

## Discussion

To the best of our knowledge, in Japan, there were no reports of any cases with both portal vein thrombosis and inferior vena cava thrombosis occurring simultaneously.

Generally, venous thrombosis is caused by the three factors of Virchow’s triad, namely, blood flow abnormality, vascular wall abnormality, and abnormal coagulation-fibrinolytic capacity. It is thought that intraperitoneal venous thrombosis is caused by blood flow disorder associated with portal hypertension caused by liver cirrhosis or Budd-Chiari syndrome, vascular wall injury caused by the spread of inflammation due to infection, surgery, or trauma, and abnormalities in the coagulation-fibrinolytic system, such as protein C, protein S, and antiphospholipid antibody syndrome.

In this case, in addition to consciousness disorder, blood test results showed a high inflammatory response, the patient was suffering from multiple organ failure (SOFA score 9), and ascites and intestinal edema were observed; therefore, treatment was started for sepsis due to intraperitoneal organ infection. It is thought that consciousness disorder improved with a decrease in serum ammonia level by fluid loading (progress table). The main causes of venous thrombosis include the aforementioned blood flow abnormality to abnormal coagulation-fibrinolytic capacity, but liver cirrhosis and Budd-Chiari syndrome were ruled out by the gastroenterology department. There was no history of abdominal surgery or trauma, and a detailed examination for abnormalities in the coagulation-fibrinolytic system was performed at the hematology department, but no notable finding was found. According to Watanabe et al., infections causing portal vein thrombosis include acute appendicitis, biliary infection, intraperitoneal abscess, and diverticulitis [1], but it is difficult to suspect them from the course and images until admission.

Treatment for venous thrombosis includes thrombolytic therapy, anticoagulant therapy, and surgical treatment. Urokinase (u-PA) and alteplase (t-PA) are used as thrombolytic therapy. The administration routes include systemic administration, administration in the superior mesenteric artery, and percutaneous transhepatic intraportal administration; however, it is said that there is no difference in clinical efficacy [1]. Heparin, danaparoid, warfarin, and AT Ⅲ formulation are used for anticoagulant therapy. In the survey results compiled by Kojima et al., danaparoid alone was most frequently administered, followed by warfarin alone, danaparoid and AT Ⅲ formulation, heparin and warfarin, and heparin. For heparin, the median daily dose was 10,000 units and the median administration duration was nine days; for danaparoid, the median daily dose was 2,500 units and the median administration duration was 14 days; for AT Ⅲ formulation, the median daily dose was 1,500 units and the median administration duration was three days [2]. In this case, anticoagulant therapy with heparin was started from the day after admission. Heparin was chosen since it is used frequently in our department. Targeting an activated partial thromboplastin time (APTT) level of 40-60 s, 10,000-25,000 units/day were given. In addition, as the AT Ⅲ formulation enhances AT activity by binding with heparin, 1,500 units were administered per day targeting an AT Ⅲ level of around 70%. In this case, as a result, intestinal necrosis occurred, requiring emergency surgery; therefore, it might have been necessary to set a high APTT level. However, according to Kojima et al., the mean heparin dose was 14,400 units for 11.1 days [2]; while in this case, the mean heparin dose was around 20,000 units for 23 days. There was no previous case to refer to for further increasing the dose, so caution must be exercised. However, studies in other countries have reported that anticoagulant therapy in patients with portal vein thrombosis does not increase the risk of bleeding [3], suggesting its advantage; therefore, it may be good to consider targeting an APTT level of 80 s, as presented by the cardiology department. In addition, many institutions are using danaparoid rather than heparin, and compared with heparin, danaparoid has higher factor Ⅹa activity, higher thrombolytic effect, and less effect on platelets, and hence the risk of bleeding is lower; thus, the use of danaparoid should also be considered. Thrombectomy as a surgical treatment is rarely performed due to injuries to the collateral circulation and frequent recurrences after thrombectomy [4]. However, if intestinal ischemia is suspected, early surgical intervention is performed as a rule [5,6]. Intestinal resection areas include methods such as blood flow abnormality resection up to thrombus-forming marginal veins, vascular wall abnormality resection of only necrotic intestines, and abnormal coagulation-fibrinolytic capacity manual removal of thrombi in the mesentery and anastomosis of healthy intestinal tracts. The postoperative recurrence rate has been reported to be 11%-9%, and mortality due to recurrence has been reported to be 37% [7-9]. Time to recurrence is 1-40 days, with a mean of 11 days [5,9]. In our case, CT on day 1 of admission revealed no notable finding in the abdomen other than intestinal edema and ascites. Contrast-enhanced CT on day 7 of illness showed that the thrombus in the superior mesenteric vein was expanding to the caudal side, and the pros and cons of thrombus retrieval were considered in the cardiovascular surgery department, the cardiology department, and the gastrointestinal surgery department. Thoracoabdominal contrast-enhanced CT on day 10 of illness (Figure 5) revealed low-contrast regions in the small bowel, but the general condition was stable, there was no elevation in the lactate level, and there lacked findings such as abdominal pain; therefore, the surgery was shelved by the gastrointestinal surgery department. It has been reported that if ascites were observed in the superior mesenteric venous thrombosis, intestinal necrosis was found in nine of 10 cases, and if thrombectomy was not performed, intestinal necrosis progressed and reoperation was required in 60% [10]; therefore, early surgical intervention should be considered.

## Conclusions

When a patient is admitted to the hospital for loss of consciousness, it can be challenging to identify intraperitoneal venous thrombosis. However, when diagnosed, it is necessary to administer anticoagulants early and determine an appropriate surgical intervention, for example, bowel resection and thrombi retrieval. There were no previous reports of extensive thrombosis from the inferior vena cava and portal vein to the superior mesenteric vein, and an appropriate and effective course of treatment was difficult to discern.
